# Supplementary material for: Capacity of Retinal Ganglion Cells Derived from Human Induced Pluripotent Stem Cells to Suppress T-Cells
Source: Int J Mol Sci. 2020 Oct 22;21(21):7831. doi: 10.3390/ijms21217831 (PMC7660053; doi:10.3390/ijms21217831)
Supplement: Supplementary file 1 [file ijms-21-07831-s001.pdf]

Supplementary materials

Fig. S1.

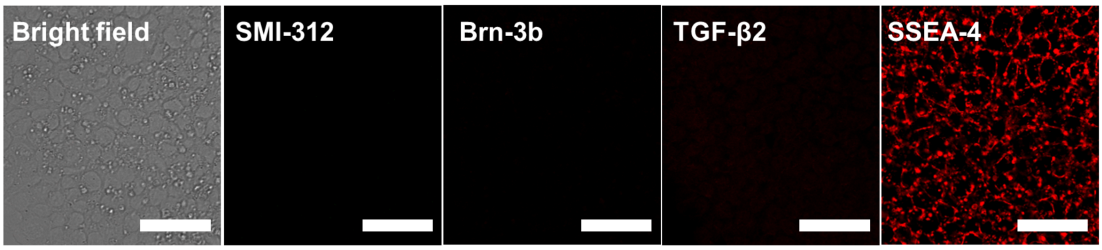

Fig. S1. Immunocytochemical staining for SMI-312, Brn-3b, TGF-β2, and SSEA-4 in iPSCs, which were used as control cells.

The iPSCs, which we used as control cells, did not stain with SMI-312, Brn-3b, or TGF-β2. On the other hand, iPSCs clearly expressed the pluripotency marker, SSEA-4. Scale bars, 50 μm.

Fig. S2.

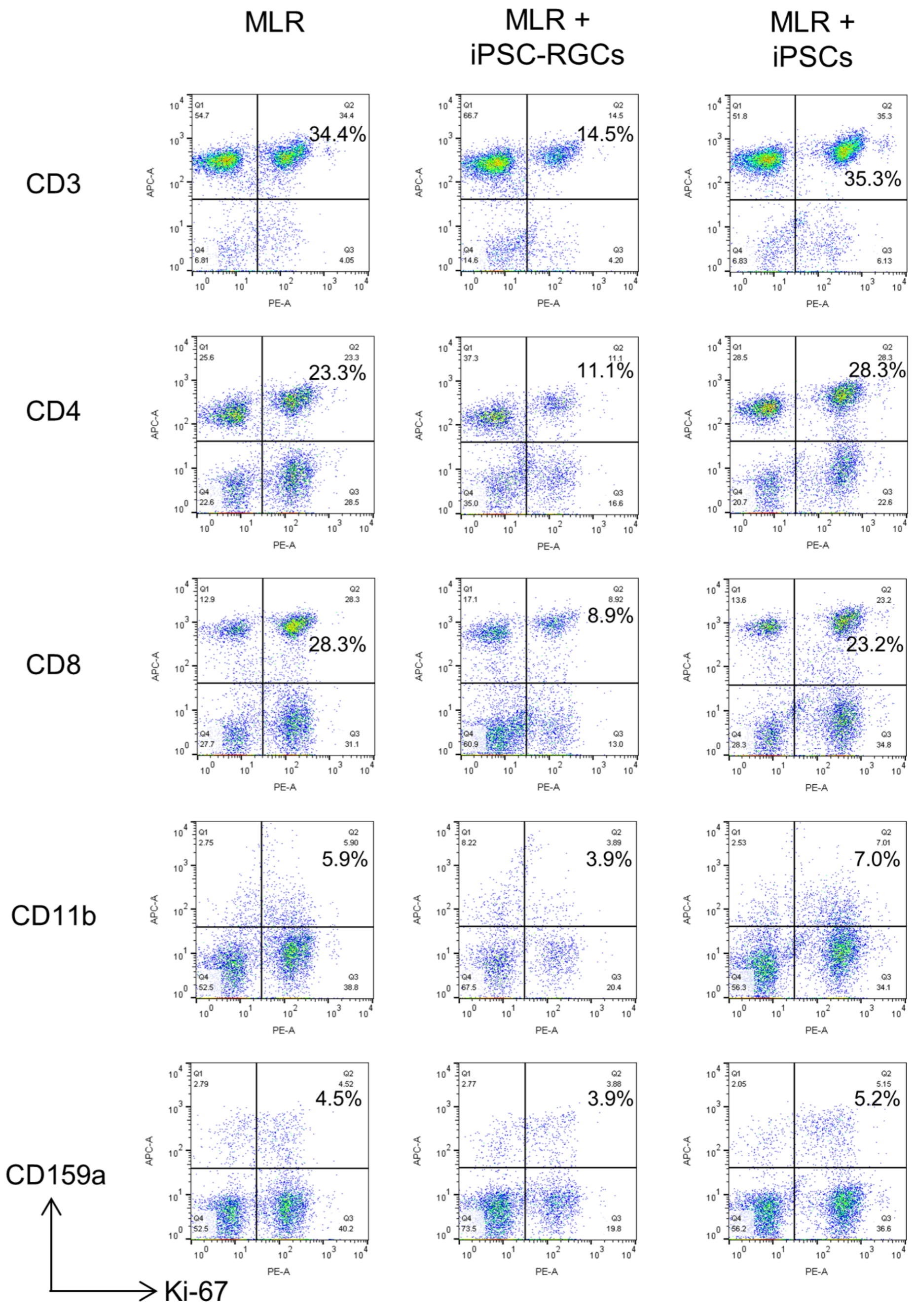

Fig. S2. Capacity of iPSC-RGCs from another donor to suppress lymphocytes proliferation. Another representative result of Ki-67 FACS proliferation analysis in the MLR assay using another RGCs line. We co-cultured MLR cells and 201B7 iPSC-RGCs (MLR:RGC ratio = 2:1) for 120 hours. iPSC-RGCs suppressed the proliferation of CD3-, CD4-, CD8-, CD11b- and CD159a-positive cells. Numbers

in the scatterplots indicate the percentage of double-positive cells for CD3, CD4, CD8, CD11b, or CD159a and Ki-67.

Fig. S3.

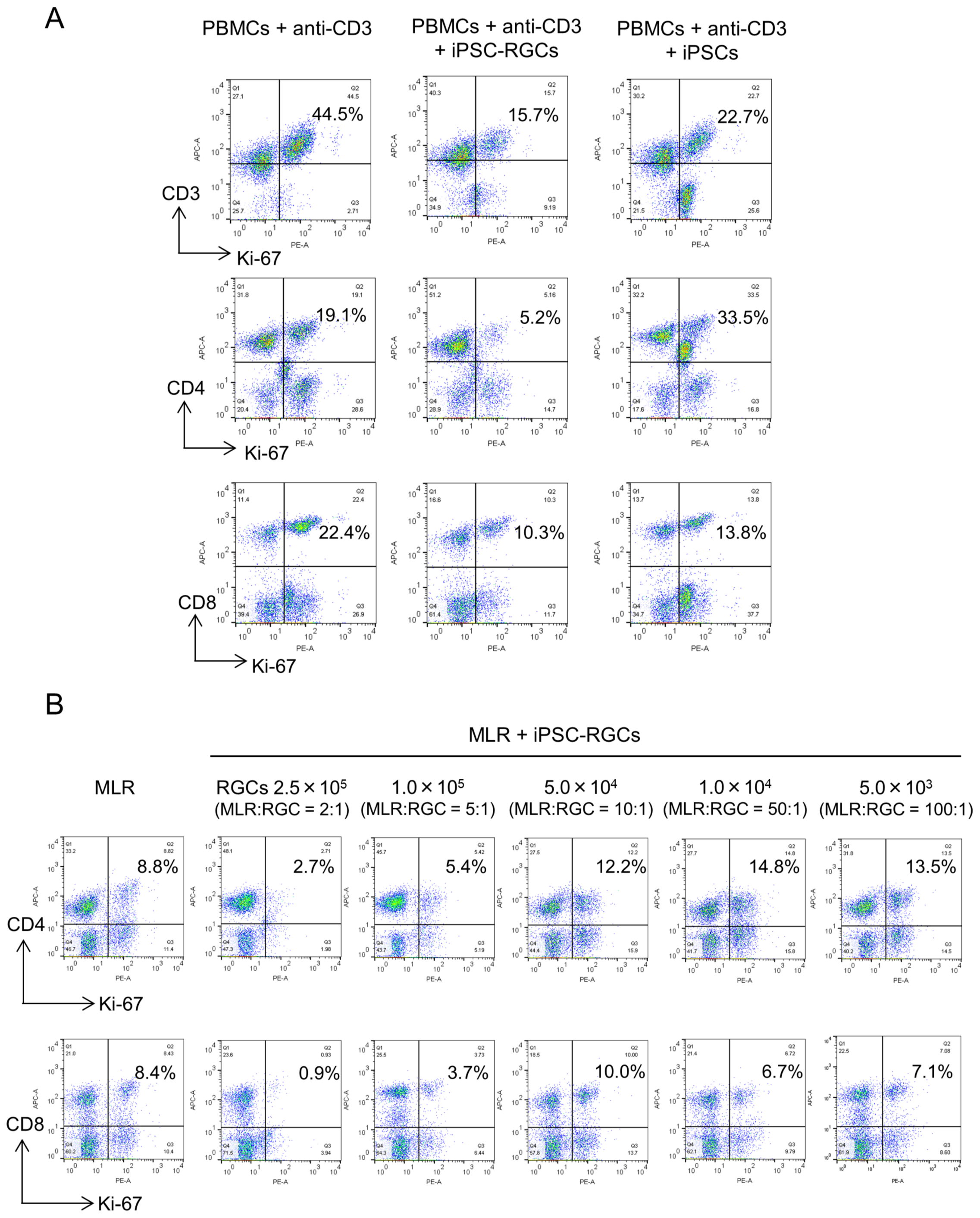

Fig. S3. Capacity of iPSC-RGCs to suppress T-cell proliferation *in vitro*.

(**A**) To confirm whether iPSC-RGCs could inhibit T-cell proliferation even when activated by anti-human CD3 agonistic antibody, we co-cultured PBMCs and iPSC-RGCs (MLR:RGC ratio = 2:1) with anti-CD3

antibody (0.1 μg/mL). After 72 hours of incubation, PBMCs were analyzed with Ki-67 FACS. We prepared iPSCs as control cells. iPSC-RGCs greatly suppressed the proliferation of CD3-, CD4-, and CD8-positive T cells. Numbers in the scatterplots indicate the percentage of double-positive cells for CD3, CD4, or CD8 and Ki-67. (**B**) To examine whether the ratio of iPSC-RGCs to MLR cells affects the immunosuppressive effects of iPSC-RGCs, we co-cultured 5×10^5 MLR cells (mixed PBMCs from healthy donors) with 5.0×10^3 to 2.5×10^5 iPSC-RGCs (MLR:RGC ratio = 100:1, 50:1, 10:1, 5:1, and 2:1). After 120 hours of incubation, Ki-67 proliferation FACS analysis was performed. As a result, 2.5 $\times 10^5$ and 1.0×10^5 iPSC-RGCs (MLR:RGC ratio = 2:1 and 5:1) inhibited CD4- and CD8-positive T-cell proliferation. On the other hand, 5.0×10^3 to 5.0×10^4 iPSC-RGCs (MLR:RGC ratio = 100:1, 50:1, and 10:1) failed to suppress T-cell proliferation. Numbers in the scatterplots indicate the percentage of double-positive cells for CD4 or CD8 and Ki-67.

Fig. S4.

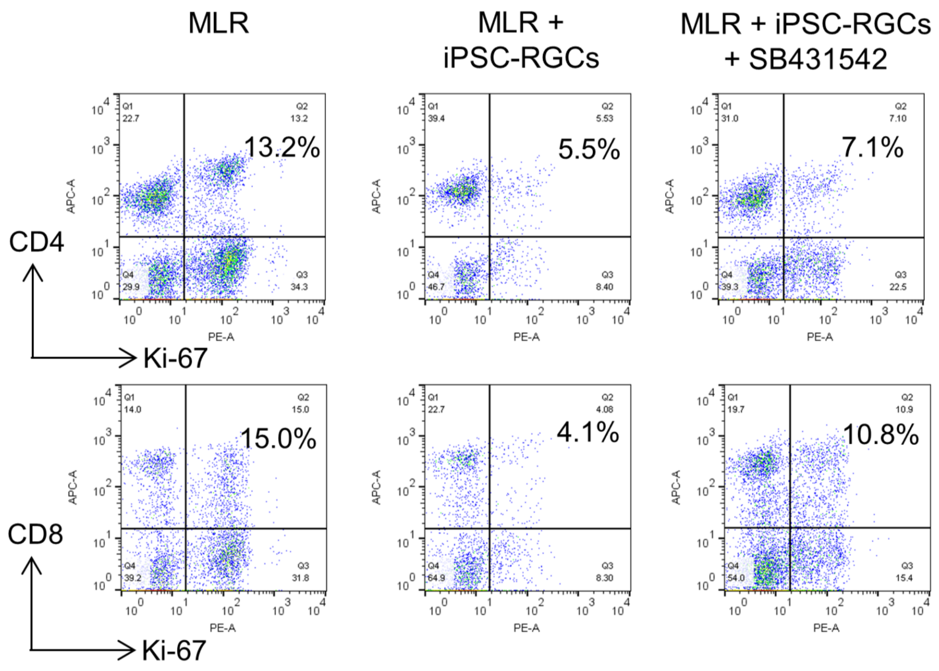

Fig. S4. Role of TGF-β in the immunosuppressive effects of iPSC-RGCs.

Another representative result of Ki-67 FACS proliferation analysis in the MLR assay with TGF-β blocking. We co-cultured MLR cells and iPSC-RGCs (MLR:RGC ratio = 2:1) while inhibiting TGF-β using SB431542 for 120 hours. In the presence of SB431542, the inhibition of T-cell proliferation by iPSC-RGCs was blocked. Numbers in the scatterplots indicate the percentage of double-positive cells for CD4 or CD8 and Ki-67.

Table S1. Antibody information

No.	Antibody	Species	Dilution	Source	Catalog #
1	Anti-human CD80 (B7-1), FITC	Mouse	5 μL/test	Thermo Fisher Scientific	11-0809-42
2	Anti-human CD86 (B7-2), FITC	Mouse	10 μL/test	BD Biosciences	555657
3	Anti-human CD274 (PD-L1), PE	Mouse	5 μL/test	Invitrogen Life Sciences	12-5983-42
4	Mouse IgG, isotype control, FITC	-	5 μL/test	R&D Systems	IC002F
5	Mouse IgG, isotype control, PE	-	5 μL/test	Thermo Fisher Scientific	IC002P
6	Anti-pan axonal neurofilament marker (SMI)	Mouse	1:100	Novus Biologicals	2-29435
7	Anti-Brn-3b	Goat	1:1000	Santa Cruz Biotechnology	SC-6026
8	Anti-TGF-β2	Rabbit	1:1000	R&D Systems	AB-12-NA
9	Mouse IgG	-	1:1000	Abcam	ab170190
10	Rabbit IgG	-	1:1000	Thermo Fisher Scientific	02-6102
11	Goat IgG	-	1:1000	Thermo Fisher Scientific	02-6202
12	Alexa Fluor 488 anti-mouse IgG (H+L)	Donkey	1:1000	Thermo Fisher Scientific	A21202
13	Alexa Fluor 546 anti-mouse IgG (H+L)	Donkey	1:1000	Thermo Fisher Scientific	A10036
14	Alexa Fluor 546 anti-goat IgG (H+L)	Donkey	1:1000	Thermo Fisher Scientific	A11056
15	Alexa Fluor 546 anti-rabbit IgG (H+L)	Donkey	1:1000	Thermo Fisher Scientific	A11040
16	Anti-human CRX	Rabbit	1:1000	Takara	M231
19	Anti-Nanog	Mouse	1:1000	Reprocell	RCAM0003P
20	Anti-SSEA-4	Mouse	1:200	Millipore	MAB4304
21	Cy3-Donkey Anti-Rabbit IgG (H+L)	Donkey	1:200	Jackson Immuno Research	711-165-152
22	Anti-human HLA class I (HLA-A, B, C)	Mouse	1:100	Thermo Fisher Scientific	14-9983-82
23	Anti-human HLA class II (HLA-DR, DP, DQ)	Mouse	1:100	BD Biosciences	555556

24	Anti-human Ki-67, PE	Mouse	3 µL/test	BioLegend	350504
25	Anti-human CD3, APC	Mouse	2 µL/test	Invitrogen Life Sciences	300412
26	Anti-human CD4, APC	Mouse	2 µL/test	Miltenyi Biotec	130-113-250
27	Anti-human CD8, APC	Mouse	5 µL/test	Thermo Fisher Scientific	170088-42
28	Anti-human CD11b, APC	Mouse	2 µL/test	Miltenyi Biotec	130-113-231
29	Anti-human CD159a (NKG2A), APC	Mouse	2 µL/test	Miltenyi Biotec	130-113-563
30	Mouse IgG1, isotype control, APC	-	2 µL/test	Miltenyi Biotec	130-113-196
31	Mouse IgG, isotype control, PE	-	3 µL/test	BioLegend	400112

Table S2. Primer sequences and probe number in quantitative RT-PCR

Gene	Forward sequence (5'-3') of primers	Reverse sequence (5'-3') of primers	Probe*
Brn-3b (POU4F2)	TATGCGGAGAGCCTGTCTTC	CTCTGGGAGACGATGTCCAC	#42
ISL1	AAGGACAAGAAGCGAAGCAT	TTCCTGTCATCCCCTGGATA	#66
RBPMS	CTGTACCCAGCGGAGTTAGC	TGCCTCAGGAGAGAAACACTG	#63
THY1	CAGAACGTCACAGTGCTCAGA	GAGGAGGGAGAGGGAGAGC	#66
GAPDH	AGCCACATCGCTCAGACAC	GCCCAATACGACCAAATCC	#60
TGF-β1	CAGCCGGTTGCTGAGGTA	GCAGCACGTGGAGCTGTA	#72
TGF-β2	CAGATGCTTCTGGATTTATGGTATT	CCAAAGGGTACAATGCCAAC	#67
TGF-β3	AAGAAGCGGGCTTTGGAC	CGCACACAGCAGTTCTCC	#38
CD3e	CAAGGCCAAGCCTGTGAC	TCATAGTCTGGGTTGGGAACA	#49

*Probe - The probe in the Roche Universal Probe Library was used for quantitative RT-PCR assay.
